# Electrophysiological correlates of associative learning in smokers: a higher-order conditioning experiment

**DOI:** 10.1186/1471-2202-13-8

**Published:** 2012-01-11

**Authors:** Marianne Littel, Ingmar HA Franken

**Affiliations:** 1 Institute of Psychology, Erasmus University Rotterdam, P.O. Box 1738, 3000 DR, Rotterdam, the Netherlands

## Abstract

**Background:**

Classical conditioning has been suggested to play an important role in the development, maintenance, and relapse of tobacco smoking. Several studies have shown that initially neutral stimuli that are directly paired with smoking are able to elicit conditioned responses. However, there have been few human studies that demonstrate the contribution of higher-order conditioning to smoking addiction, although it is assumed that higher-order conditioning predominates learning in the outside world. In the present study a higher-order conditioning task was designed in which brain responses of smokers and non-smokers were conditioned by pairing smoking-related and neutral stimuli (CS1_smoke _and CS1_neutral_) with two geometrical figures (CS2_smoke _and CS2_neutral_). ERPs were recorded to all CSs.

**Results:**

Data showed that the geometrical figure that was paired with smoking stimuli elicited significantly larger P2 and P3 waves than the geometrical figure that was paired with neutral stimuli. During the first half of the experiment this effect was only present in smokers whereas non-smokers displayed no significant differences between both stimuli, indicating that neutral cues paired with motivationally relevant smoking-related stimuli gain more motivational significance even though they were never paired directly with smoking. These conclusions are underscored by self-reported evidence of enhanced second-order conditioning in smokers.

**Conclusions:**

It can be concluded that smokers show associative learning for higher-order smoking-related stimuli. The present study directly shows the contribution of higher-order conditioning to smoking addiction and is the first to reveal its electrophysiological correlates. Although results are preliminary, they may help in understanding the etiology of smoking addiction and its persistence.

## Background

Classical conditioning has been suggested to play an important role in the development, maintenance, and relapse of drug use [e.g., [1-5]]. Classical conditioning theory predicts that with repeated drug use, drug-related stimuli or contexts (conditioned stimuli, CS) become associated with drug intake (unconditioned stimulus, UCS), and consequently, in the course of time, these stimuli acquire motivational significance and evoke conditioned drug responses or cue reactivity, such as subjective craving, drug seeking behaviors, or changes in physiological measures [e.g., [6-9]]. Once the learning process has taken place and the CS are able to elicit the conditioned drug responses, the CS can be paired with new neutral stimuli or contexts, which will also acquire associative strength and elicit conditioned drug responses or cue reactivity. This process is called second-order conditioning (higher-order conditioning; CS-CS learning) and can lead to unlimited sequences of associations that presumably contribute to drug-seeking in real world environments [10-12].

Classical conditioning requires the storage of a neural representation of the associations between conditioned incentive stimuli and the conditioned responses they elicit. These associations are both reflexive and cognitively-mediated, and, accordingly, multiple neural mechanisms of learning and memory seem to be involved [13]. Potential substrates are the amygdala, which is thought to be implicated in emotional processing involving discrete cues; the hippocampus, which is assumed to play a major role in contextual learning; the striatum, which mediates procedural and habit learning; and cortical systems such as the anterior cingulate cortex and the orbitofrontal cortex that have more regulatory and general information processing functions [14]. Under normal circumstances, these neural substrates are involved in behaviors that are needed for survival, such as obtaining food, sex, and other natural rewards. However, after repeated drug use they are recruited or 'hijacked' by the drugs of abuse, producing maladaptive behavioral and cellular changes that maintain addiction [1,2].

A variety of animal studies provides evidence for the role of associative learning in drug use. With regard to nicotine addiction, animals increase self-administration of nicotine in the presence of stimuli that were previously paired with nicotine administration and they display preferences for contexts that were previously paired with nicotine administration [e.g., [15,16]]. In addition, several animal studies have demonstrated that environmental cues can maintain and reinstate drug seeking behaviors and drug administration [e.g., [16,17]]. In addition to first-order nicotine conditioning, second-order nicotine conditioning has also been demonstrated in animals [e.g., [18,19]]. For example, Goldberg et al. [18] showed that monkeys press a lever at high rates under a second-order schedule of reinforcement in which lever pressing produces a visual stimulus that is occasionally predictive of nicotine administration.

In human research, it has been shown that smokers show increased physiological reactions (e.g., heart rate, skin conductance) and report higher levels of craving following the presentation of smoking-related stimuli than following the presentation of non-smoking stimuli [see [9,20,21] for reviews]. However, it can only be assumed that these responses reflect prior classical conditioning; only studies in which UCS-CS associations are formed *ad hoc*, i.e., only studies in which initially neutral stimuli are paired with smoking within an experimental paradigm can be decisive on this issue. Lazev et al. [22] were the first to directly support the conditioning hypothesis in smokers. It was demonstrated in their study that after pairing with smoking, visual, olfactory, and auditory stimuli increase pulse rate and self-reported cigarette craving. In several other studies it was shown that smokers report higher levels of craving when exposed to a cue or context that has been paired with the occurrence of smoking than when exposed to a cue or context paired with the nonoccurrence of smoking [8,23,24]. Moreover, it was shown that smokers show greater approach tendency towards smoking stimuli that were presented in the presence of a cue predicting nicotine intake [25], that smokers exhibit greater preparatory physiological responses, i.e., skin conductance and facial electromyographic responses, greater salivary responses and enhanced EEG beta power in the presence of abstract cues paired with smoking [26-28] and that they attend selectively to discriminative cues that signal the availability of tobacco-smoke reinforcement [e.g., [29,30], and see [31] for an overview].

Second-order conditioning in smoking addiction has been less extensively studied in humans. However, some studies have been conducted that can be considered second-order conditioning studies. In these studies, neutral cues were paired with the expectancy of winning or losing cigarettes (instead of really obtaining cigarettes or real smoking). The neutral cues that were associated with the expectancy of winning cigarettes elicited greater attentional bias, enhanced drug-seeking behavior and consumption, and more pleasurable mood states than cues that were associated with the expectancy of losing cigarettes [e.g., [30,32]]. However, no studies have been conducted in which neutral cues were paired with conditioned smoking-related cues.

Since conditioned cue-reactivity appears to play such an important role in the continuation of smoking behavior and relapse after periods of abstinence, and since smoking has so many deleterious effects on health, investigating associative learning in smoking addiction into greater detail is of major relevance. Little is known about the neural correlates of classical conditioning in smoking addiction and even less is known about the contribution of higher-order conditioning to smoking addiction. According to Gewirtz and Davis [11], studying the latter is particularly important, since only little human learning involves the direct pairing of stimuli or contexts with powerful, unconditioned reinforcers. Higher-order conditioning presumably predominates learning in the outside world.

One way to study the neural correlates of associative learning in smokers is by measuring event-related potentials (ERP) using electroencephalography (EEG) techniques. ERPs are electrophysiological brain responses to internal or external stimuli, which consist of several time-locked components that convey a certain amplitude magnitude. They are particularly suited to study differences in cognitive processing, because the magnitude of the amplitude can provide us with information about the extent of engagement, whereas the locations can teach us more about the neurobiological generators. It is assumed that enhancement of the later components of the ERP, i.e., the P3 and the Late Positive Potential (LPP) reflects enhanced motivated attention for the stimuli presented [33-37].

In studies of cognitive processes and biases in addiction, these later components of the ERP are of particular relevance. Several studies show that the P3 and the LPP are larger in drug users than in controls in response to drug-related stimuli compared to neutral stimuli [e.g., [38-42]]. In smokers, a centro-frontally distributed enlargement of P3 and LPP amplitudes has been found in response to smoking cues relative to matched neutral cues, whereas non-smokers show no difference in P3 and LPP amplitudes to both stimulus categories [43-46]. These ERP studies among smokers provide evidence for the assumption that smoking-related cues capture attentional resources and that smokers exhibit enhanced motivated attention towards smoking-related stimuli. This assumption is underlined by the fact that in most studies of drug addiction P3 and LPP amplitudes have been found to correlate with subjective craving [see [47] for an overview].

Recently, Franken et al. [48] used ERP methodology in order to study classical conditioning of emotional stimuli. They found an increased P3 in response to initially neutral stimuli (CS) that predicted the occurrence of emotional pictures compared to CS that predicted the occurrence of neutral pictures. These results demonstrate that conditioning processes, including higher-order conditioning processes (emotional pictures have acquired motivational relevance during life), can be measured with ERPs, and, moreover, that the P3 is a suitable index of acquired motivational relevance.

The present study was conducted in order to examine higher-order learning processes associated with smoking addiction and its electrophysiological correlates. Based on the experimental paradigm used by Franken et al. [48], a second-order smoking conditioning task utilizing ERP methodology was designed. Smoking-related and neutral stimuli (pictures; CS1_smoke _and CS1_neutral_) were paired with two geometrical figures (CS2_smoke _and CS2_neutral_). Both smokers' and non-smokers' ERPs in response to the CS1 and the preceding CS2 were recorded throughout the experiment. The abovementioned measure, i.e., enhanced motivated attention for stimuli reflected by increased P3 amplitudes in response to CS2, was used as an outcome measure for conditioning to have taken place. The rationale is that if relatively neutral and meaningless figures become capable of differentially eliciting increased P3 amplitudes (which are associated with enhanced motivated attention and preference for stimuli) after pairing with meaningful and motivationally relevant pictures, associative learning must have taken place.

We predict that the geometrical figures will become associated with the pictures and will elicit enhanced P3 amplitudes. In smokers, we expect this P3 enhancement to be more evident for CS2_smoke _than for CS2_neutral_, whereas we expect to find no differences between stimuli in non-smokers. Furthermore, we predict that smokers will rate the CS2_smoke _as more positively valenced, more arousing, and eliciting more subjective craving than the CS2_neutral_. No rating differences (valence, arousal) are expected in non-smokers.

Although the P3 is the best-described ERP index of attention, several earlier ERP components have been associated with attention processing and shown to co vary with intrinsic motivational properties of stimuli, including the P1, N1, and P2 [49-51]. These components were investigated in an exploratory manner and differential enhancement of these components was also regarded as indication for associative learning.

## Methods

### Participants

Thirty smokers (5 males, 25 females) and 31 non-smokers (5 males, 26 females) participated in the present study. They were recruited from the Erasmus University Rotterdam (the Netherlands) and received either financial compensation or course credit for participation. Non-smokers (mean age 20.5 years, SD = 1.9) were included if they had smoked fewer than 5 cigarettes in their lifetimes (mean 1.1, SD = 1.5). Smokers (mean age 21.9 years, SD = 3.0) were eligible if they smoked at least 10 cigarettes per day on average (mean = 15.6, SD = 4.2). Smokers had a mean score of 4.4 (SD = 1.9) on the Dutch version of the Fagerström Test for Nicotine Dependence [FTND; 52], which suggests that they had low to medium levels of dependence. Furthermore, they had a mean carbon monoxide (CO) level of 12.6 parts per million (Ppm; SD = 7.8), which differed significantly from non-smokers' CO level (mean Ppm = 1.1, SD = 1.1), *t*(59) = 8.17, *p *< 0.001. The present study was approved by the local ethics committee of the Institute of Psychology. All participants provided informed consent.

### Stimuli and experimental paradigm

**Figure 1:**
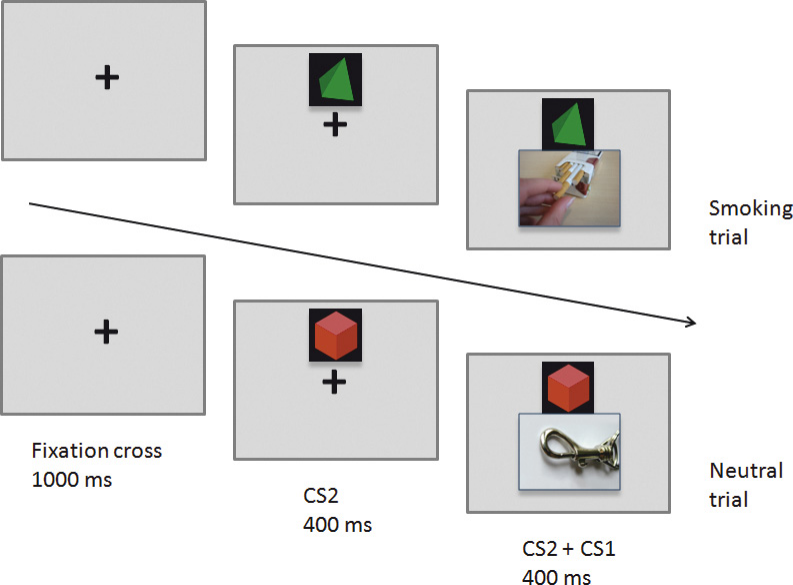
**Schematic representation of the experimental paradigm**.

Forty smoking-related pictures (people smoking or holding cigarettes and smoking-related objects) and 40 neutral pictures selected from the International Affective Picture System [IAPS; 53] served as CS1. These pictures were paired with two geometrical figures, i.e., a green pyramid and a red cube, which served as CS2. Each trial started with a fixation cross which was presented for 1000 ms. Subsequently, one of the two geometrical figures was presented in the upper half of the screen with a duration of 800 ms. After 400 ms of CS2 presentation, a smoking-related (CS1_smoke_) or neutral (CS1_neutral_) picture was added in the center of the screen. This CS1 remained visible for 400 ms. Inter trial interval was 500 ms. See Figure 1 for a schematic representation of the experimental paradigm. For each participant, the same CS2 was always paired with the same CS1 type (e.g., the cube was always paired with the smoking pictures). Pairing combinations were counterbalanced across subjects. In total there were 160 trials: 80 CS2-neutral trials and 80 CS2-smoking trials. All CS2-CS1 pairs were presented in a random order. Each CS1 was presented 4 times. After 40 CS2-CS1 pairs, participants received 15 second breaks.

### Procedure

Smokers were instructed to abstain from smoking for at least one hour prior to the experiment in order to avoid direct effects of nicotine on ERP signals. They were told that this was absolutely necessary and that it would be checked with a breath analyzer. After obtaining written informed consent, participants filled out several questionnaires on demographics, smoking history, and smoking dependence (smokers). After completion, participants were seated in a comfortable chair which was positioned in a light and sound-attenuated room. After the attachment of the electrodes, participants proceeded to a non-invasive CO Ppm estimate utilizing the EC50 Micro III Smokerlyzer^® ^(Bedfont Scientific, Medfort, NJ, USA), a portable device that measures breath carbon monoxide levels. Participants were instructed to sit still, to focus on the fixation cross in the center of the screen, to blink in between stimulus presentations, and to carefully watch the stimuli without employing distracting thoughts. Furthermore, they were explicitly told to search for an association between the figures and the pictures. After picture viewing, participants rated the CS2_smoke _and CS2_neutral _on valence and arousal properties utilizing a 10 cm Visual Analogue Scale (VAS). In addition, smokers rated both CS2 on their capacity to elicit cigarette craving. Finally, all participants were asked to fill out what they thought the association was between the two geometrical figures and the pictures.

### Self-report measures

All participants reported sex and age. Additionally, smokers reported smoking duration and number of cigarettes per day, whereas non-smokers reported number of cigarettes smoked in their lifetimes. Smoking dependence was measured with the Dutch version of the Fagerström Test of Nicotine Dependence [FTND; 52]. The questionnaire consists of six items and has good reliability [52]. All participants rated the conditioned geometrical figures on valence and arousal properties by means of a 10 cm VAS. Smokers also rated the conditioned figures on craving properties, again by means of a 10 cm VAS. Since it has been hypothesized that contingency awareness is necessary for learned motivation in humans [see [31] for an overview], contingency awareness was tested by asking participants to write down the associations between the geometrical figures and the pictures.

### Electroencephalogram (EEG) recording and signal processing

**Figure 2:**
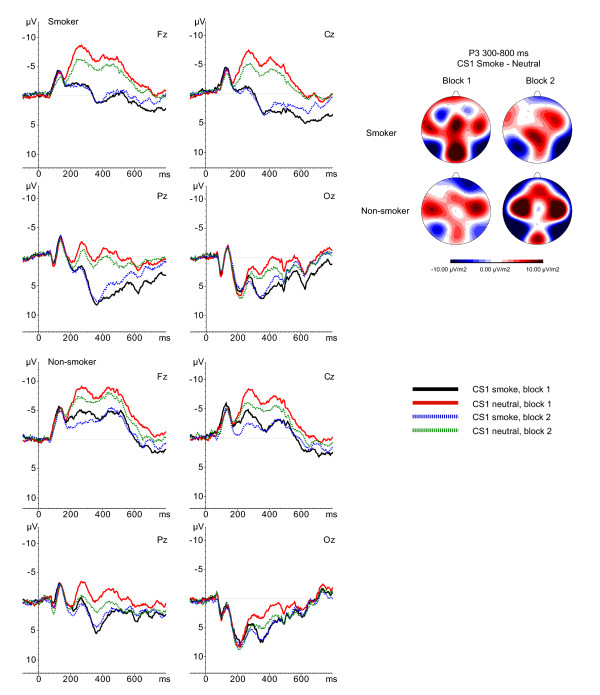
**Smokers' (upper) and non-smokers' ERPs (lower) in response to CS1_smoke _and CS1_neutral _in Block1 and Block 2**. Current Source Density (CSD) maps represent differences in activity between CS1_smoke _and CS1_neutral _in the 300-800 ms timeframe (P3).

**Figure 3:**
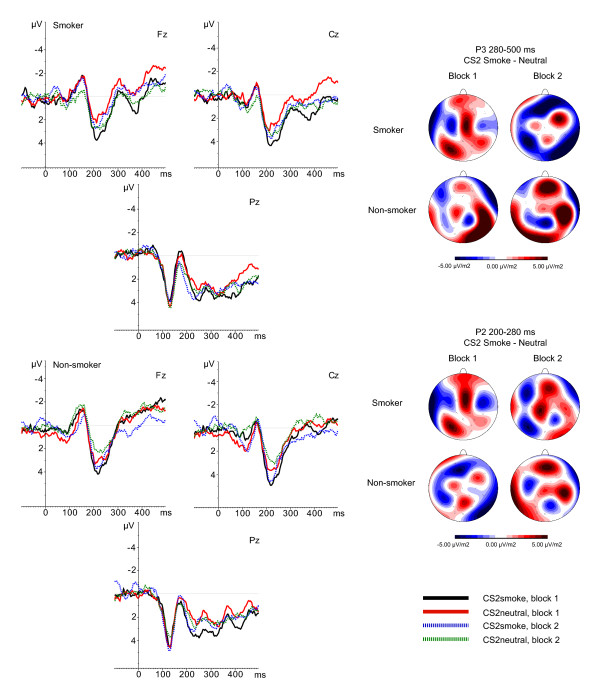
**Smokers' (upper) and non-smokers' ERPs (lower) in response to CS2_smoke _and CS2_neutral _in Block 1 and Block 2**. Current Source Density (CSD) maps represent differences in activity between CS2_smoke _and CS2_neutral _in the 280-500 ms timeframe (P3) and the 200-280 ms timeframe (P2).

The electroencephalogram (EEG) was recorded using a BioSemi Active-Two amplifier system (BioSemi, Amsterdam, the Netherlands) from 34 scalp sites [International 10/10 system; 54, 55] using active Ag/AgCl electrodes mounted in an elastic cap. Six additional electrodes were attached. Two electrodes were attached to the left and right mastoids, two to the outer canthi of both eyes (horizontal electro-oculogram; HEOG), and two to the infraorbital and supraorbital regions of the eye (vertical electro-oculogram; VEOG). Both an active electrode (CMS - common mode sense) and a passive electrode (DRL - driven right leg) were used to comprise a feedback loop for amplifier reference. Signals were recorded online with a low pass filter of 134 Hz and digitized with a 512 Hz, 24-bit A/D converter. Offline, the EEG signals were referenced to the mathematically linked mastoids and EEG and EOG were filtered with a band pass of 0.01-80 Hz (phase shift-free; 24 dB/octave slope). CS1 data were segmented in epochs of 900 ms, including 100 ms pre-stimulus baseline, whereas CS2 data were segmented in epochs of 600 ms, including 100 ms pre-stimulus baseline. Ocular correction [56] was applied and epochs containing an EEG signal exceeding ± 75 μV were excluded from the average. After baseline correction, epochs were averaged across trials and overall grand averages were obtained for the two CS1 conditions (CS1_smoke _and CS1_neutral_) and for the first and the last 40 trials of the two CS2 conditions (CS2_smoke _and CS2_neutral_). The resulting ERP waves were visually inspected and appeared to correspond well with ERP waves usually reported in response to visual stimuli. Regarding the CS1, a clear P3 was identified in the 300-800 ms time window (see Figure 2). Regarding the CS2, a P3 component was observed in the 280-500 ms timeframe (see Figure 3). In addition, P1, N2, and P2 components were identified in the 100-150 ms, the 150-200 ms, and the 250-280 ms timeframe, respectively. For all components elicited by the CS1 and CS2, mean activity (area measurement) was computed per group and stimulus category. Brain Vision Analyzer (Brain Products, Germany) was used for all offline EEG analyses.

### Analyses

For each component ERP effects were assessed by performing repeated-measurement analyses of variance (ANOVAs). Based on current source density (CSD) maps for differences in brain activity between CS1 conditions (Figure 2) 10 electrodes of interest were selected, i.e., FC1, Fz, FC2, C3, Cz, C4, CP1, Pz, Cp2, and Oz. For analyzing P2 and P3 components elicited by CS2 conditions three midline electrode sites (Fz, Cz, Pz) were selected (Figure 3). Analyses of P1 and N1 components were restricted to occipital electrode sites, i.e., PO3, O1, Oz, O2, PO4. Group (smokers versus non-smokers) served as the between-subjects factor. CS1 stimulus type (neutral versus smoking-related), block (first block, second block), and electrode site served as within-subjects factors in the ANOVA on CS1, and CS2 stimulus type (neutral versus smoking-related), block (first block, second block), and electrode site served as within-subjects factors in the ANOVAs on CS2. Arousal, valence, and craving ratings of the geometrical figures were tested using two 2 (stimulus) × 2 (group) repeated-measurement ANOVAs (arousal and valence) and an independent t-test (craving). Because of missing data, two participants were excluded from this analysis.

Greenhouse-Geisser correction was applied to all ANOVAs (uncorrected df's are reported). All significant effects and effects showing trends towards significance were further analyzed using pairwise comparision post-hoc tests. An alpha-level of 0.05 was used for all statistical tests.

## Results

### First-order Conditioned Stimuli (pictures; CS1)

P3

**Figure 4:**
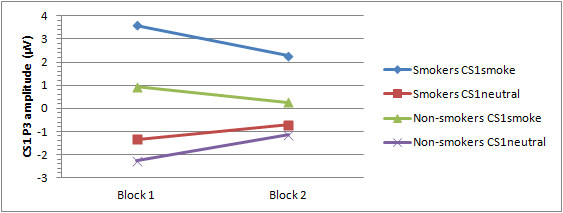
**Mean P3 amplitudes for smokers and non-smokers per Block and CS1**.

See Figures 2 and 4 for mean P3 amplitudes per group, CS1 and block. A significant main effect for CS1 was observed *F*(1,59) = 94.17, *p *< 0.001. Smoking pictures elicit larger P3 amplitudes than neutral pictures across participants. In addition, a significant CS1 × Block interaction was found, *F*(1,59) = 12.02, *p *= 0.001. Post-hoc tests revealed that the P3 amplitude in response to CS1_smoke _is larger during the first 40 trials than during the last 40 trials (*p *= 0.009), whereas there is a trend for the P3 in response to CS1_neutral _to be larger during the last 40 trials compared to the first 40 trials (*p *= 0.064). However, in both blocks the CS1_smoke _elicit larger P3 amplitudes than the CS1_neutral _(both *p*'s < 0.001). Furthermore, a significant CS1 × Group effect, *F*(1,59) = 6.57, *p *= 0.013, was found. Post-hoc comparisons revealed that smokers respond with significantly larger P3 amplitudes to CS1_smoke _than to CS1_neutral _(*p *= 0.001), whereas non-smokers show no amplitude difference between the two CS1 (*p *= 0.239). In addition, a trend towards a significant CS1 × Electrode × Group effect, *F*(3,177) = 2.05, *p *= 0.064, was observed. Post-hoc tests showed larger P3 amplitudes for CS1_smoke _in smokers relative to controls at all electrodes (all *p*'s < 0.01), except for Oz.

### Second-order Conditioned Stimuli (geometrical figures; CS2)

P3

**Figure 5:**
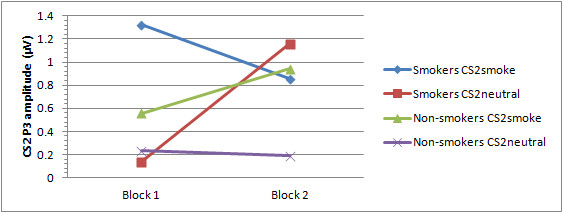
**Mean P3 amplitude for smokers and non-smokers per Block and CS2**.

See Figures 3 and 5 for mean P3 amplitudes per group, CS2 and block. First of all, the main effect for CS2 showed a trend towards significance, *F*(1,59) = 0.075, with CS2_smoke _showing larger amplitudes than CS2_neutral _across all participants and blocks indicating a conditioning effect for all participants in response to figures associated with smoking pictures. Furthermore, a significant CS2 × Block × Group interaction effect was observed, *F*(1,59) = 4.45, p = 0.039. Post-hoc comparisons revealed that during the first block, smokers' P3 amplitude in response to the CS2_smoke _is significantly larger than their P3 amplitude to the CS2_neutral _(*p *= 0.019), whereas non-smokers show no difference between P3 amplitude in response to the CS2_smoke _and CS2_neutral _during the first 40 trials (*p *= 0.502). Neither smokers, nor non-smokers show differences in P3 amplitude in response to CS2_smoke _and CS2_neutral _during the second block (smokers: *p *= 0.557; non-smokers: *p *= 0.142). Furthermore, although not significant, smokers show trends to respond with an enlarged P3 to CS2_neutral _than non-smokers during the second block (*p *= 0.095) and an enhanced P3 to CS2_neutral _in the second block compared to the first block (*p *= 0.078). Furthermore, a significant CS2 × Block × Electrode × Group interaction was found, *F*(3,177) = 6.26, *p *= 0.003. Post-hoc comparisons showed that in the first block, smokers display a more positive P3 in response to CS2_smoke _than to CS2_neutral _at Fz (*p *= 0.028), Cz (*p *= 0.018) and Pz (*p *= 0.091), whereas non-smokers display a larger P3 to CS2_smoke _than to CS2_neutral _during the second block (Fz: *p *= 0.078, Cz: *p *= 0.046). In addition, during the second block smokers' P3 in response to CS2_neutral _becomes greater than non-smokers' P3 in response to CS2_neutral _at Cz (*p *= 0.046). Moreover, although the conditioning effect for CS2_smoke _does not change from the first to the second block, smokers show enhancement of P3 amplitudes for CS2_neutral _in second block as compared to the first block at Fz (*p *= 0.052) and Cz (*p *= 0.064). Non-smokers, show the opposite pattern, i.e., no changes of the conditioning effect for neutral cues between the first and the second block, but an enhancement of P3 amplitudes for CS2_smoke _in the second block as compared to the first block at Fz (*p *= 0.030).

### Early components

**Figure 6:**
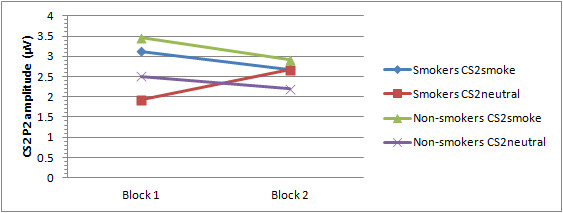
**Mean P2 amplitude for smokers and non-smokers per Block and CS2**.

No significant main or interaction effects were found for the P1. For the N1, a significant main effect was found for Block, *F*(1,59) = 5.98, *p *= 0.017, indicating that N1 amplitudes are larger during block 1 than during block 2. No other main or interaction effects were observed for this component. For the P2 a significant main effect for CS2 was observed, *F*(1,59) = 4.28, p = 0.043. Figures associated with smoking pictures elicit larger P2 amplitudes than figures associated with neutral figures across participants and blocks. In addition, a significant CS2 × Block × Electrode × Group interaction was found, *F*(2,118) = 8.54, *p *= 0.001. Post-hoc comparisons revealed that in block 1 smokers show enlarged P2 amplitudes in response to CS2_smoke _as compared to CS2_neutral _at Fz (*p *= 0.029) and Cz (*p *= 0.049) electrodes. During this first block non-smokers also show enlarged P2 amplitudes in response to CS2_smoke _as compared to CS2_neutral _but in contrast to the effect found in smokers, this effect not present at Fz and Cz, but at Pz (*p *= 0.040). During the second block no significant main or interaction effects were found. See Figures 3 and 6 for mean P2 amplitudes per group, CS2 and block.

### Behavioral measures

**Figure 7:**
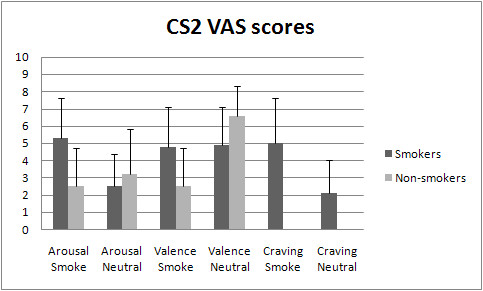
**Smokers' and non-smokers' mean self-reported arousal and valence ratings and smokers' mean self-reported craving ratings of the CS2_smoke _and CS2_neutral _measured with a 10 cm Visual Analogue Scale (VAS)**. Error bars represent standard deviations.

All participants were aware of the CS2-CS1 relation. Smokers' craving ratings (self-reported craving elicited by CS) were significantly higher for the CS2_smoke _than for the CS2_neutral_, *t*(28) = 5.98, *p *< 0.001. Furthermore, on both arousal and valence judgments of the geometrical figures significant CS2 × Group interactions were found, respectively *F*(1,57) = 28.79, *p *< 0.001 and *F*(1,57) = 18.80, *p *< 0.001. Post-hoc tests showed that smokers rate the CS2_smoke _as significantly more arousing than non-smokers (p < 0.001). They also find the CS2_smoke _more positive than do non-smokers, (p < 0.001). Smokers and non-smokers do not differ in arousal judgment of the CS2_neutral _(*p *= 0.191). However, non-smokers find the CS2_neutral _significantly more pleasant than smokers (*p *< 0.001). See Figure 7 for smokers' and non-smokers' mean valence, arousal, and craving scores.

## Discussion

The aim of the present study was to examine higher-order classical conditioning processes associated with smoking addiction by employing a direct cortical measure, i.e., brain activity as measured by ERPs. Brain responses of smokers and non-smokers were conditioned by pairing smoking-related and neutral stimuli (CS1_smoke _and CS1_neutral_) with two geometrical figures (CS2_smoke _and CS2_neutral_). All subjects were consciously aware of the CS2-CS1 associations. ERPs were recorded to both CS1 and preceding CS2.

With regard to the CS1, results from the present study replicate the previously observed finding that smokers exhibit a processing bias for smoking-related stimuli [43,45,46]. At frontal, central and parietal sites, P3 components of the ERP were larger in response to smoking cues than in response to neutral cues for smokers compared to non-smokers. This implies that our smoking stimuli were suitable to serve as CS1 in the current study. Furthermore, it was observed that smoking cues elicited larger P3 amplitudes than neutral cues across participants, indicating that smoking cues in general captured more attention than neutral cues.

With regard to the CS2, we expected that through second-order associative learning the neutral figures would become associated with the pictures that followed them and would therefore start to elicit equal conditioned responses, i.e., motivated attention and preference as reflected by increased ERP amplitudes. We predicted this ERP enlargement to be more evident for CS2_smoke _than for CS2_neutral _in smokers, whereas we expected to find no differences between stimuli in non-smokers. Although all clearly discernable ERP components (P1, N1, P2, and P3) were analyzed, P3 components were of special interest since they have been associated with motivated attention for the cues presented [33-37] and because previous studies show that they are enlarged in smokers in response to smoking related pictures that are suggested to have acquired motivational significance through prior first-order classical conditioning [43,45,46]. Results showed that P3 amplitudes were enlarged in response to CS2_smoke _as compared to CS2_neutral _across participants. This implies an overall conditioning effect for geometrical figures associated with smoking cues. This finding is in accordance with electrophysiological responding to the CS1 (i.e., smoking cues capturing more attentional resources than neutral cues in general). Comparable results were obtained for the P2. P2 amplitudes were enlarged in response to CS2_smoke _as compared to CS2_neutral _across participants. Although the P2 is reported less often than the P3 in picture processing, there are indications that this earlier component is also sensitive to automatic attention capture and could be modulated by stimulus valence [49-51].

Furthermore, in line with our primary hypotheses, data from the present study showed that in smokers, the CS2_smoke_, i.e., the geometrical figure that was paired with smoking stimuli, elicited significantly greater P3 amplitudes than the CS2_neutral _during the first half of the experiment, whereas no differences between CS2 conditions were found in non-smokers. Similarly, the CS2_smoke _was shown to elicit larger P2 amplitudes than CS2_neutral _in smokers but not in non-smokers. These results suggest that smokers, compared to non-smokers, show more enhanced associative learning for smoking cues than for neutral cues even though these cues were never paired directly with an UCS (i.e., smoking). Furthermore, it underscores the idea that addiction affects basic learning and memory systems, and that their neural substrates, normally involved in obtaining more conventional goals, are recruited by the drugs of abuse [2,14].

Besides the electrophysiological evidence, we also observed self-reported evidence of enhanced second-order conditioning in smokers: smokers reported more cue-elicited craving for the CS2_smoke _compared to the CS2_neutral_. Furthermore, smokers found the CS2_smoke _more arousing and more pleasurable than non-smokers. Besides, they rated the CS2_neutral _as less pleasurable than non-smokers. As can be observed in Figure 7, the valence differences between smokers and non-smokers with regard to the CS2_smoke _were partly driven by the finding that non-smokers rate the CS2_smoke _as less pleasurable or more aversive than smokers, since smokers find the CS2_smoke _and the CS2_neutral _equally pleasurable. Yet, the group differences remain and indicate that smokers and non-smokers engage in different learning patterns.

Direct first-order smoking conditioning has already been demonstrated in several previous studies. In these studies it was shown that smokers report more craving and show greater approach bias, attentional bias and physiological responses in response to cues paired with the presence of smoking than in response to cues paired with the absence of smoking [8,22-25]. Although no explicit reference was made to it, second-order conditioning has already been demonstrated in several studies in which neutral cues were paired with the expectancy of winning or losing cigarettes. The neutral cues that were associated with the expectancy of winning cigarettes elicited greater attentional bias, enhanced drug-seeking behavior and consumption, and more pleasurable mood states than cues that were associated with the expectancy of losing cigarettes [e.g., [30,32]]. The present study is the first to demonstrate direct second-order conditioning in smoking addiction by combining neutral stimuli with conditioned smoking pictures.

During the course of the experiment, the group-specific conditioning effects of the present study seem to reverse; during the last half of the experiment smokers' P3 amplitudes in response to the CS2_neutral _increased relative to the first half of the experiment, whereas non-smokers' P3 amplitudes to the CS2_smoke _increased relative to the first half experiment. Moreover, smokers' P3 amplitude in response to CS2_neutral _became significantly larger than that of non-smokers across all electrodes, whereas non-smokers' P3 amplitude to CS2_smoke _became significantly larger than that of smokers at Pz. With regard to the P2, all significant conditioning effects found during the first half of the experiment disappeared during the second half (see Figures 5 and 6 for a visual representation of these results).

Although great interpretive caution is warranted, it appears that non-smokers show a slowly progressing learning curve for CS2 _smoke_, i.e., stimuli that are more attentively processed (see results on CS1). Smokers, on the other hand, show a steeper learning curve for CS2 _smoke _during the first trials of the experiment, suggesting an initially enhanced associative learning for smoking cues as compared to non-smokers. However, this enhanced smoking-related associative learning declines after a certain amount of time, and seems to be replaced by a delayed conditioning for the neutral cues.

There exist several possible explanations for this finding. First of all, second-order conditioning is intrinsically weaker than first-order conditioning and appears typically to be transient [11]. It is argued that after a small number of trials second-order learning reaches a maximum and starts to decline with further training. Gewirtz and Davis [11] posit that this is caused by the development of conditioned inhibition; the CS2 becomes a signal for the nonoccurrence of reinforcement and therefore inhibits the elicitation of conditioned responses. In line with this is the hypothesis that drug use expectancy is necessary for learned motivation in humans [see [31] for an overview]. Although second-order conditioning develops faster than conditioned inhibition, the latter is the strongest of the two phenomena. Therefore, a plausible explanation for decrement of the conditioning effect for smoking cues during the second part of the experiment might be that after some time smokers lose their interest in the cues paired with smoking stimuli, since they predict no real smoking and subsequent reinforcement and start focusing on the cues paired with neutral stimuli instead. Evidence for this explanation is provided by the results from the CS1 by Block interaction analyses, which showed that P3 amplitudes in response to smoking pictures were larger during the first trials than during the last trials, whereas there was a trend for the P3 in response to neutral pictures to be larger during the last trials compared to the first trials. However, because scores on the valence, arousal, and craving VAS, which were collected at the end of the conditioning session, still show a clear subjective conditioning effect, it can be argued whether smokers really lost their interest in the CS2_smoke _during the second block due to conditioned inhibition or the absence of contingencies. A plausible alternative explanation could be merely fatigue or boredom, since the experiment conveys many repetitions of the same figures and pictures.

## Conclusions

The present findings suggest that smokers and non-smokers show associative learning during a higher-order conditioning experiment in response to neutral cues that are paired with smoking-related stimuli as measured with ERP indices of attention. Furthermore, results indicate that this associative learning is more pronounced in smokers than in non-smokers during the first half of the experiment. These effects are not only found on the electrophysiological level, as reflected by enlarged P2 and P3 components of the ERP, but are also self-reported. Therefore, the results indicate that for smokers neutral cues that are paired with motivationally relevant smoking-related stimuli gain more motivational significance, at least for a short period of time, even though they were never paired directly with an UCS.

We acknowledge that we only investigated the effects of explicit conditioning as we instructed participants to pay attention to the presented contingencies. Therefore, the conclusions are limited by the fact that they only pertain to explicit conditioning. Although several studies have shown that in addictive behaviors implicit processes may have limited value in conditioning effects [31], the present paradigm could be employed to further elucidate this role on the neurophysiological level. Furthermore, it must be noted that there was an overrepresentation of female participants. Because there were no gender differences between groups, these could not have accounted for the observed group differences. In addition, we assume that the functional meaning attributed to P2 and P3 responses can be safely applied to electrophysiological responding to CS2. We acknowledge that there exists no certainty regarding this issue. However, some evidence for this assumption can be derived from the study by Franken et al. (2011). In this study an increased P3 was observed in response to neutral stimuli that predicted the occurrence of emotional pictures compared to neutral stimuli that predicted the occurrence of neutral pictures, indicating that the P3 is a suitable index of acquired motivational relevance.

This is the first study to directly show the contribution of higher-order conditioning to smoking addiction. Furthermore, it is the first study to reveal the electrophysiological correlates of higher-order conditioning in smoking. Replication studies are warranted, ideally using a design in which actual smoking is paired with certain neutral cues, which are in turn paired with other neutral cues.

Although results are preliminary, they may help in understanding the etiology of smoking addiction and its persistence. Craving and relapse might not be triggered by concrete cues and contexts only, but also, or predominantly, by more complex and divergent cues and contexts which do not necessarily have intrinsic motivational value, but have motivational value that is acquired through the processes of higher-order conditioning.

## Authors' contributions

ML designed and carried out the higher-order conditioning experiment, performed the statistical analysis, created the figures and wrote manuscript. IF supervised the process and was involved in revising the manuscript critically for important intellectual content. All authors read and approved the final manuscript.

## Acknowledgements and funding

This study was supported by a grant of the Netherlands Organization for Scientific Research (NWO; VIDI grant number 016.08.322). The funding organization had no role in design and conduct of the study, neither in data analysis and interpretation. No approval of the manuscript was required from the funding organization.

We would like to thank Marina Koers for her assistance with data collection and data management and Maartje Luijten for her valuable comments on previous versions of the manuscript.
